# The association between caregiver burden, distress, psychiatric morbidity and healthcare utilization among persons with dementia in Singapore

**DOI:** 10.1186/s12877-021-02014-2

**Published:** 2021-01-19

**Authors:** Jue Hua Lau, Edimansyah Abdin, Anitha Jeyagurunathan, Esmond Seow, Li Ling Ng, Janhavi Ajit Vaingankar, Siow Ann Chong, Mythily Subramaniam

**Affiliations:** 1 Research Division, Institute of Mental Health, Buangkok Green Medical Park, 10 Buangkok View, Singapore, Singapore; 2 Department of Rheumatology & Immunology, Singapore General Hospital, SingHealth, Singapore, Singapore

**Keywords:** Dementia, Dementia caregivers, Caregiver burden, Healthcare utilization, Caregiver psychiatric morbidity, Responsive behaviours

## Abstract

**Background:**

Caregivers of persons with dementia (PWD) face high caregiving burden, distress related to responsive behaviours, and psychiatric morbidity. The present paper examines how these are associated with healthcare utilization of the PWD in Singapore.

**Methods:**

The data of 399 caregiver-PWD dyads were extracted from a national cross-sectional survey. PWD completed the Client Service Receipt Inventory, which provided information on their healthcare utilization (i.e. emergency service use, hospital admission, length of stay in hospital, and number of outpatient visits) within a frame of 3 months. The Zarit Burden Interview  (ZBI), Neuropsychiatric Inventory Questionnaire (NPI-Q), and Self Reporting Questionnaire (SRQ-20) were administered to caregivers. Information on severity of dementia, physical multimorbidity of the PWD, household composition, and caregivers’ sociodemographic characteristics such as age, gender, and education were collected. Variables significantly associated with healthcare utilization in the univariate analyses were selected and included in the final regression models. Emergency service use and hospital admission were investigated using logistic regression analyses, whilst negative binomial models were utilized for length of stay in hospital and number of outpatient visits.

**Results:**

After adjusting for significant correlates such as dementia severity and multimorbidity, only caregiver distress from responsive behaviours was positively associated with emergency room utilization, while caregiver burden was positively associated with length of hospital stay in the final regression model. Psychiatric morbidity was associated with healthcare utilization outcomes at the univariate level but did not reach statistical significance in final models.

**Conclusion:**

The study identifies caregiver variables associated with the healthcare utilization of PWD. Policy makers and healthcare professionals should provide interventions to ease burden and distress amongst caregivers of PWD.

**Supplementary Information:**

The online version contains supplementary material available at 10.1186/s12877-021-02014-2.

## Background

Dementia refers to a broad category of neurological diseases that cause impairment of cognition, emotion, language, motivation, and daily functioning [1], often leading to significant detrimental effects on the wellbeing of caregivers of persons with dementia (PWD) [2]. It was estimated that in 2016, there were 43.8 million PWD globally, with a 117% increase from 20.2 million in 1990 [3]. The World Health Organization has estimated this figure will rise to 152 million by 2050 [4]. Within Singapore alone, 10% of the older adults aged 60 years and above suffered from dementia based on a nationwide epidemiological survey conducted in 2013 [5]. In 2019, adults aged 60 years and above consisted 21.4% of Singapore’s population [6], and this percentage has been estimated by the United Nations to double to 40.1% by 2050  [7]. This means that in the future, even with prevalence remaining unchanged, the number of people living with dementia will likely increase. Therefore, dementia is not just a medical problem, but an economic and societal issue that concerns the individual, family and the state.

PWD generally require a high level of care, which is often provided by informal or family caregivers, and this often comes at a high cost. A study conducted amongst caregivers of PWD in Singapore estimated that the net cost of informal care for PWD was SG$39,053 per person per year, leading to a total net cost of SG$1.76 billion in 2015 [8]. Apart from financial hardship, caregivers often have to struggle with the physical, emotional, and social strain that accompanied caring for a PWD. The strain of care-giving is also associated with high caregiving burden, psychiatric morbidity, and poorer physical health [9–11], ultimately resulting in poorer quality of life. The minor psychiatric disorders, mostly depression and anxiety, which are common and lead to significant burden of disability, are referred to as psychiatric morbidity in the current paper [11, 12]. Within Singapore, a nationwide cross-sectional study of 693 pairs of informal caregivers and older adults estimated that caregivers of PWD or older adults with any responsive behaviours were 2.3 to 2.5 times more likely to have psychiatric morbidity [11]. Responsive behaviours, known also as Behavioural and Psychological Symptoms of Dementia (BPSD), is an umbrella term that represents a heterogenous group of clinical phenomena experienced by the PWD, such as disturbed emotions, mood, perception, thought, motor activity, and altered personality traits [13]. Extant literature has shown that responsive behaviours are associated with high levels of distress in PWD and caregivers, as well as increased healthcare utilization [14–16].

Much of the research conducted in Western societies indicates that PWD have a higher utilization of emergency departments, and higher rates of hospital admissions [17, 18], and outpatient visits [19] than those without dementia. A systematic review and meta-analysis indicated that reasons for hospital admissions for PWD include falls, respiratory, renal and gastrointestinal infections, as well as cardiovascular and psychiatric conditions [20]. Within Singapore, multimorbidity (i.e. two or more co-occurring chronic conditions) among adults aged 60 and above was associated with substantially higher healthcare utilization and social care costs [21]. When admitted into acute hospitals, PWD are at higher risks of functional decline [22] and mortality [23]. While many studies have focused on how characteristics of the PWD are related with healthcare utilization to identify opportunities for improvement, few studies have examined how caregiver characteristics are associated with their care recipient’s healthcare utilization. For example, Lang et al. [24] found that amongst PWD who were hospitalized via emergency department, caregiver burden was associated with prolonged institutionalizations. Moreover, a study examining PWD in an acute tertiary hospital in Singapore found that caregiver stress was associated with increased length of stay [25]. A recent longitudinal study found that depression and caregiver burden in caregivers of PWD was significantly associated with their care recipient’s emergency department use [26]. All in all, the evidence suggests that caregiver characteristics may be associated with the healthcare utilization of PWD. This is especially important since the caregiver serves as a gatekeeper to the healthcare utilization of the PWD, and therefore the threshold for seeking medical attention for the patient likely depends upon the characteristics of the caregiver [14].

Due to the dearth of literature in this area, the present study seeks to examine how caregiver burden, psychiatric morbidity, distress arising from responsive behaviours, are associated with the PWD’s healthcare utilization such as emergency services, hospital admissions, hospital length of stay, and number of outpatient visits.

## Methods

### Sample

The dataset utilized for the present study was derived from the Well-being of Singapore Elderly (WiSE) study that was conducted between August 2012 to December 2013 [5]. The WiSE study was a single-phase, cross-sectional survey of Singapore residents (citizens and permanent residents) aged 60 years and above. Ethical approval of the WiSE study was obtained from the National Healthcare Group Domain Specific Review Board as well as the Singhealth Centralized Institutional Review Board. Residents aged 60 years and above were randomly selected using a disproportionate stratified sampling from a national administrative database that contains details about the age, gender, ethnicity and addresses of all residents. The study oversampled certain minority populations, such as residents aged 75 and above, and those of Malay and Indian ethnicity in order to ensure sufficient sample size for the reliability of parameter estimates achieved within these population subgroups. Residents who were selected were sent notification letters inviting them to participate in the study and that an interviewer would visit their address for an interview. Residents did not need to respond with permission for the interviewers to visit their residence, but they could choose to opt out by contacting the hotline stated on the notification letters. Interviewers then visited the residences to schedule an interview on the same day or on a later date. Individuals who had moved out of the country, or were unable to complete the interview in either English, Mandarin, Malay, Tamil, or Chinese Dialects (Hokkien, Teochew, and Cantonese), were excluded from the study.

In addition, each older adult was asked to identify one family member or friend, who according to the older adult, “knew the older adult best”. Informants were eligible for the study if they were Singapore residents, and aged 21 years and above. Informants did not need to be residing with the older adult but had to be someone who would be able to provide the clearest and most detailed account of the older adult’s health conditions and service use. Approximate time spent with the older adult was used as a criterion for deciding the best informant in the situation where there were several co-resident family members involved in the care or decision-making of the older adult. Based on three broad questions that assessed the care needs and care-arrangements of the older adult, informants were classified as either a ‘hands-on’ (directly provided care) or ‘organizational’ (made care arrangements and decisions) caregiver.

Informed consent was obtained from all residents and their caregivers prior to the interview. For older adults who were not cognitively capable of providing informed consent, written consent was obtained from a legal presentative or next-of-kin. A total of 2565 older adults completed the study, yielding a response rate of 65.6%. Of these respondents, 2421 had an informant who completed the interview as well.

The survey-weighted (weighted by age and ethnicity) prevalence of dementia was estimated to be 10% in the WiSE study [5]. In all 399 of 2565 (15.55%) of older adults met the criteria for dementia within the sample. Data from these older adults and their informants, who were also their informal caregivers, were extracted from the WiSE study dataset and served as the sample for the present study. Informants for this group of older adults will henceforth be referred to as “informal caregivers” within the present paper.

### Measures

#### Client service receipt inventory

Information regarding healthcare utilization was collected via an adapted version of the Client Service Receipt Inventory [21], which contains questions about specific community, hospital, and informal care services utilized during the 3 month period prior to the interview. Both care recipients and caregivers were asked whether the care recipients had: i) visited an emergency department in the last 3 months, ii) been admitted for at least a 24 h stay to any hospital in the last 3 months, iii) how many nights were spent if they were admitted (i.e. length of hospital stay), iv) the number of visits in the last 3 months made to polyclinics (primary care clinics), v) restructured hospitals, and vi) general practitioners and vii) private hospitals. The total number of outpatient visits in this study was obtained by summing the number of visits made to polyclinics, restructured hospitals, and general practitioners and private hospitals.

#### Caregiver distress related to responsive behaviours

Responsive behaviours, or BPSD, were assessed with the Neuropsychiatric Inventory Questionnaire (NPI-Q [27];) that was administered to the caregivers by trained lay interviewers. The NPI-Q is a 12 item questionnaire that assesses 12 behavioural disturbances that may occur in neuropsychiatric disorders: delusions, hallucinations, agitation or aggression, depression or dysphoria, anxiety, elation or euphoria, apathy or indifference, disinhibition, irritability or lability, motor disturbance, night time behaviour/sleep, and appetite. The NPI-Q first screens for the presence or absence of each symptom in the last month, and for each present symptom, the caregiver proceeds to rate the severity of the symptom on a 3-point scale (i.e. Symptom Severity; 0 = mild, 3 = severe), as well as the associated impact of the symptom on them on a 5-point scale (i.e. Caregiver distress arising from responsive behaviours; 0 = not distressing at all, 5 = extreme or very severe). Total scores for the Symptom Severity and Caregiver Distress related to responsive behaviours scales were obtained by summing their respective individual scores for each item. Only the Caregiver Distress arising from responsive behaviours scale was utilized since only caregiver characteristics were of interest in the present study. The internal consistency of the scale was high in the present sample (Cronbach α = 0.85).

#### Psychiatric morbidity

Psychiatric morbidity was assessed using the World Health Organization’s Self-Reporting Questionnaire (SRQ-20; 28), which detects the presence of non-psychotic symptoms such as depression, anxiety, and psychosomatic complaints, over the past 2 weeks. Consisting of 20 items which must be answered either as yes or no, the questionnaire was administered to caregivers by the trained interviewer. When all items are totalled, the SRQ-20 yields a total score ranging from 0 to 20, with higher scores reflecting higher psychiatric morbidity. The SRQ-20 possesses reasonable to strong criterion validity [28] and its many translations which have been used around the world display compelling evidence for strong reliability and validity in samples of caregivers of older adults [29–31]. In the present sample, the internal consistency of the SRQ-20 was found to be high (Cronbach α = 0.88).

#### Caregiver burden

The Zarit Burden Interview (ZBI [32]) is a 22-item questionnaire that was employed to measure the subjective burden of caregivers of adults suffering from dementia. Each item is rated on a 5-point Likert scale (0 = never, 4 = nearly always present), and examines impairments on the caregiver’s health, psychological well-being, finances, and social life, which result from them taking care of the care recipient. A total score is obtained by summing all items, with higher scores indicating higher caregiver burden. The ZBI has shown to have strong internal consistency, test-retest reliability, and construct validity in a sample of family caregivers in Singaporean [33]. The internal consistency was found to be high in the present study (Cronbach α = 0.93).

#### Multimorbidity and severity of dementia of PWD

Information regarding the chronic physical conditions of the PWD was collected using a chronic conditions checklist [21]. Respondents were asked whether they had any of the following chronic conditions: High blood pressure; Heart trouble (including heart attack, angina, heart failure and valve disease); Stroke; Transient Ischemic Attacks (TIAs); Diabetes; Depression; Arthritis or Rheumatism; Chronic obstructive pulmonary disease (COPD); Breathlessness or asthma; and Cancer. Responses to the checklist were first grouped into three groups: “no chronic physical conditions”, “at least one chronic physical condition”, and “multimorbidity”. Multimorbidity was defined as the coexistence of two or more of these chronic physical conditions at the same time [34]. Following descriptives analyses, it appeared that no respondents in the “no chronic physical conditions” group endorsed using the emergency room or were admitted to hospital in the last 3 months. Since this group would be dropped from subsequent logistic regression models as there were zero responses in the outcome for comparison, the “no chronic physical conditions” and “at least one chronic physical condition” groups were subsumed into a single category: “No Multimorbidity” to aid in retention of data and interpretation.

Severity of dementia was assessed via Clinical Dementia Rating [35], which was administered via a semi structured interview to the subject and caregiver. The CDR is based on a scale of 0–3: (0) no dementia, (0.5) questionable dementia, (1) mild dementia, (2) moderate dementia, and (3) severe dementia. For further regression analyses, groups were collapsed into: i) no/questionable dementia, ii) mild dementia, iii) moderate/severe dementia.

#### Household composition and income of PWD

Household composition was obtained by having respondents and their caregivers list the ages and genders of all co-residents and their relationships to the respondent. This information was sorted into four categories: i) Lives alone, ii) lives with spouse only, iii) with spouse and others, iv) with others. For regression analyses, the “lives alone” and “lives with spouse only” categories were subsumed into a single group. Information on whether the caregiver lived with the participant was also captured. Income of participant was obtained by asking respondents whether they received any income, benefits, pensions or allowances.

Sociodemographic information of caregivers such as age, gender, education level was also collected.

### Statistical analysis

All statistical analyses were conducted using Stata version 15. For descriptive statistics, frequencies and percentages were calculated for categorical variables, while means, standard deviations, 25th percentile, median, and 75th percentile values were presented for continuous variables. Due to the limited sample size, variables were first examined using univariate chi-square/Mann-Whitney-U/Kruskal-Wallis analyses. Significant variables at univariate analyses were thus selected and included in the final model of the regression analyses. Two separate logistic regression analyses were conducted to examine the variables predicting emergency room use and hospital admissions in the last 3 months.

**Fig. 1:**
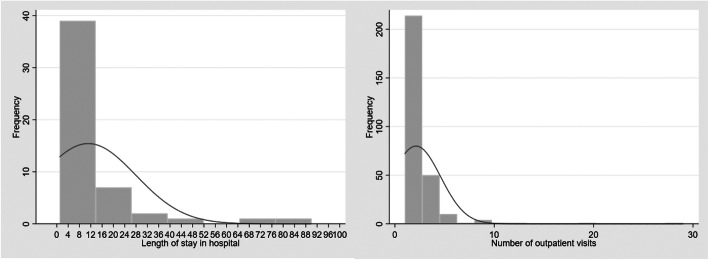
Left - Frequency distribution of variable length of hospital stay in last 3 months (in days). Right – Frequency distribution of variable number of outpatient visits in last 3 months

As displayed in Fig. 1, the count variables, length of stay in hospital and number of outpatient visits are naturally skewed distributions. Therefore, two modelling techniques that were appropriate for skewed data were specified to examine the variables associated with of length of hospital stay and number of outpatient visits: Poisson regression and negative binomial regression. In order to select the best statistical model to analyse the variables associated with length of hospital stay, several goodness-of-fit indices were utilized. Firstly, a Pearson goodness-of-fit test (χ^2^ (40) = 746.1, *p* < 0.001) and deviance goodness-of-fit test (χ^2^ (40) = 423.7, *p* < 0.001), indicating poor model fit. A likelihood-ratio test of alpha indicated that the negative binomial model describes the data better than a Poisson regression model (χ^2^ (1) = 303.2, *p* < 0.001). Furthermore, as displayed in Table 1, the mean length of hospital stay was 12.8, with a S.D. of 20.6, providing evidence highlighting the occurrence of overdispersion. In addition, the two models were also compared using Akaike’s Information Criterion (AIC) and Bayesian Information Criterion (BIC) to determine better model fit, with lower values indicating better fit. The Poisson model had a log-likelihood of − 289.4, AIC of 584.7, and BIC of 590.0. In contrast, the negative binomial model had a log-likelihood of − 137.8, AIC of 283.6, and BIC of 290.6. Therefore, the negative binomial model was selected based based on a better fit. Regarding the Poisson model examining the number of outpatient visits, although the deviance goodness-of-fit test was not significant (χ^2^ (237) = 265.6, *p* = 0.10), the Pearson goodness-of-fit test was significant (χ^2^ (237) = 376.3, *p* < 0.001), indicating that the model fit might not be optimal. Additionally, the significant likelihood-ratio test of alpha (χ^2^ (1) = 20.28, *p* < 0.001) demonstrated that the negative binomial model would be able to describe the data better than a Poisson model. Moreover, AIC and BIC values showed that the negative binomial model was a better fit (Poisson log-likelihood = − 427.4., AIC = 860.9, BIC = 871.3; Negative Binomial log-likelihood = − 417.3, AIC = 842.6, BIC = 856.5). Hence, the negative binomial regression model was selected.

**Table 1:** Characteristics of the sample (*n* = 399)

Categorical variables				n		%	
Age group of caregivers							
21 to 34 years				19		4.76	
35 to 49 years				95		23.81	
50 to 64 years				206		51.63	
65 years and above				78		19.55	
Gender of caregivers							
Female				270		67.67	
Male				128		32.08	
Caregiver living with participant							
No				66		16.54	
Yes				332		83.21	
Caregiver Education							
None/Minimal				40		10.03	
Completed primary				88		22.06	
Completed secondary				166		41.60	
Completed tertiary				103		25.81	
Chronic physical conditions of PWD							
None				62		15.54	
At least one				91		22.81	
Multimorbidity (i.e. two or more)				246		61.65	
Clinical dementia rating of PWD							
No/Questionable dementia				109		27.32	
Mild dementia				188		47.12	
Moderate/Severe dementia				102		25.56	
Participant receives income/ benefits/ pension/allowance							
No				171		42.86	
Yes				225		56.39	
Household composition							
Lives alone				10		2.51	
With spouse only				21		5.26	
With spouse and others				126		31.58	
With others				235		58.90	
Emergency Room Use in last 3 months							
No				356		89.22	
Yes				43		10.78	
Hospital Admission in last 3 months							
No				347		86.97	
Yes				52		13.03	
**Continuous variables**	n	Mean	S.D.	P25	Median	P75	Range
Caregiver Distress from Responsive Behaviours (NPI-Q)	386	3.19	6.08	0	0	4	0–48
Psychiatric Morbidity (SRQ-20)	388	2.47	3.55	0	1	3	0–20
Zarit Burden Interview (ZBI)	328	16.99	14.09	6	14	25	0–88
Length of Hospital stay in last 3 months	51	11.12	16.78	3	6	10	1–90
Number of outpatient visits in last 3 months	283	2.12	2.47	1	1	2	1–29

Frequency counts and percentages may not tally to 399 or 100% due to missing data

## Results

### Sociodemographic characteristics of the sample

Sociodemographic characteristics of the 399 caregiver-PWD dyads are presented in Table 1. Majority of the caregivers were female (67.7%), aged 50 to 64 years (51.6%). The mean age of the caregivers was 55.1 (SD 11.8), with a range of 21 to 88 years. A large proportion of PWD had multimorbidity (*n* = 246, 61.7%), and had mild dementia (*n* = 188, 47.1%).

### Emergency service utilization and hospital admission

Chi-square and Mann-Whitney-U analyses indicated that only multimorbidity, severity of dementia, psychiatric morbidity of caregiver, and caregiver distress from responsive behaviours were significantly associated with emergency service utilization at the univariate level (Additional file 1: Appendix A). For hospital admissions, only multimorbidity, severity of dementia, psychiatric morbidity, caregiver distress from responsive behaviours, and caregiver burden were significant correlates (Additional file 1: Appendix B). These variables were selected and included in final logistic regression models which are displayed in Table 2.

**Table 2:** Final model results for the logistic regression analyses of variables associated with emergency room use and hospital admission

	Emergency Room Use			Hospital Room Admission		
	O.R.	95% CI	*p*	O.R.	95% CI	*p*
Multimorbidity of PWD						
No Multimorbidity	ref			ref		
Has Multimorbidity	**4.26**	1.60–11.33	**0.004**	**3.00**	1.19–7.55	**0.02**
Clinical dementia rating (CDR)						
No/Questionable dementian	ref			Ref		
Mild dementia	2.22	0.86–5.78	0.10	2.44	0.94–6.36	0.07
Moderate/Severe dementia	1.58	0.53–4.68	0.41	1.64	0.55–4.85	0.38
Caregiver Distress from Responsive Behaviours (NPI-Q)	**1.07**	1.02–1.13	**0.003**	1.04	0.99–1.10	0.14
Psychiatric morbidity of caregiver (SRQ-20)	1.00	0.91–1.10	0.97	0.93	0.83–1.04	0.21
Caregiver burden (ZBI)^a^				1.03	1.00–1.06	0.08

Variables included in final logistic regression model were determined by a series of univariate chi-square and mann-whitney-U analyses (Additional file 1: Appendix)

^a^Caregiver burden was not included in the final logistic regression for emergency room use as it was not a significant correlate at the univariate level

*O.R.* Odds ratio; 95% confidence interval of odds ratio

Bold print highlights statistically significant odds ratio

Results from the final logistic regression model indicated that PWD with multimorbidity had higher odds of utilizing emergency service than those with no multimorbidity (OR: 4.3, 95% CI: 1.6–11.3, *p* = 0.004). Higher caregiver distress arising from responsive behaviours was also associated with higher emergency service use amongst PWD (OR: 1.1, 95% CI: 1.0–1.1, *p* = 0.003).

Only multimorbidity was significantly associated with higher likelihoods of hospital admissions in the final logistic regression model (OR: 3.0, 95% CI: 1.2–7.5, *p* = 0.02).

### Length of stay in hospital and number of outpatient visits

Univariate analyses indicated that only caregiver distress from responsive behaviours and caregiver burden were significantly associated with length of stay in hospital (Additional file 1: Appendix C). Multimorbidity and caregiver burden were significant correlates of the number of outpatient visits at the univariate level (Additional file 1: Appendix D). The final negative binomial regression models examining the variables associated with length of hospital stay and number of outpatient visits are displayed in Table 3.

**Table 3:** Results from the negative binomial regression analyses of variables associated with length of hospital stay and number of outpatient visits within the last 3 months

	Length of Hospital Stay^a^			Number of outpatient visits^b^		
	I.R.R	95% CI	*p*	I.R.R	95% CI	*p*
Caregiver burden (ZBI)	**1.03**	1.00–1.05	**0.02**	1.01	0.99–1.01	0.06
Psychiatric morbidity of caregiver (SRQ-20)^a^	0.98	0.87–1.09	0.66			
Multimorbidity of PWD^b^						
No multimorbidity				**ref**		
Has Multimorbidity				**1.52**	1.20–1.93	**0.001**

Variables included in final logistic regression model were determined by a series of univariate Kruskal-wallis and negative binomial analyses (Additional file 1: Appendix)

^a^Psychiatric morbidity of caregiver was not included in the final negative binomial model for number of outpatient visits as it was not a significant correlate at the univariate level

^b^Multimorbidity of PWD was not included in the final negative binomial model for number of outpatient visits as it was not a significant correlate at the univariate level

*I.R.R* Incidence rate ratio, *95% CI* 95% confidence interval of incidence rate ratio

Bold print highlights statistically significant incidence rate ratios

Caregiver burden was significantly associated with higher number of days spent by PWD in the hospital (IRR: 1.03, 95% CI: 1.0–1.1, *p* = 0.02). Those with multimorbidity had higher number of outpatient visits as compared to those with no multimorbidity (IRR: 1.5, 95% CI: 1.2–1.9, *p* = 0.01).

## Discussion

The present study aimed to examine the association between caregiver burden, psychiatric morbidity, distress due to responsive behaviours and healthcare utilization. The final regression models only included variables significant at the univariate level, and this included significant variables related to the PWD, such as multimorbidity, and severity of dementia. Multimorbidity emerged as an important correlate of healthcare utilization in PWD. As posited by previous studies, multimorbidity has been showed to have higher healthcare utilization and costs in both older adult populations [21, 36] and in patients with dementia [37].

At the univariate level, caregiver burden was significantly associated with hospital admissions, length of hospital stay, and number of outpatient visits. Caregiver psychiatric morbidity was associated with emergency room visits, hospital admissions, and length of hospital stay. Caregiver distress arising from responsive behaviours was associated with emergency room use and hospital admissions. However, after adjusting for significant correlates such as dementia severity and multimorbidity, only caregiver distress from responsive behaviours was positively associated with emergency room utilization, while caregiver burden was positively associated with length of hospital stay in the final regression models. Nevertheless, these present results regarding distress and burden are in line with extant literature showing that caregiver characteristics are important correlates of healthcare utilization of PWD. For example, a study of 58 PWD found that caregiver distress arising from responsive behaviours was associated with increased risk of emergency visits and hospitalization of the PWD [14]. Taniguchi et al. [38] found that caregiver burden caused by behavioural and psychological symptoms of dementia was a significant predictor of a longer hospital stay. Similarly, Lang et al. [24] revealed that caregiver burden was associated with prolonged hospital stays for PWD who were admitted via an emergency department. Studies also indicated that higher caregiver burden was associated with higher risks of long-term institutionalization of PWD into hospitals [39, 40]. It is possible that due to the distress from responsive behaviours and burden from caregiving, caregivers were unable to provide adequate care for the PWD, leading to increased healthcare utilization. On the other hand, studies have indicated that caregiver burden and distress are associated with increased likelihoods of neglect of older persons [41], and may even lead to potentially harmful behaviours such as verbal and/or physical abuse [42, 43]. Therefore, it might be plausible that PWDs may utilize healthcare services more due to poor quality of care at home. Furthermore, caregivers of PWD have been known to experience high levels of emotional exhaustion [44], and overwhelmed caregivers may result in premature institutionalization or increased healthcare utilization of the PWD [45].

It is of interest to note that psychiatric morbidity was not significantly correlated with any of the health-care utilization outcomes in the final regression models. A recent study by Guterman et al. [26] revealed that caregiver depression and burden were positively associated with PWDs’ emergency room usage amongst 663 caregiver-patient dyads, even after adjusting for severity of dementia, and number of comorbidities. Psychiatric morbidity within the present study was a single continuous variable based on depression, anxiety, and psychosomatic symptoms. This may have not fully captured the association between caregiver depression and healthcare utilization. Future studies would do well to separately assess depression and anxiety of caregivers using well-validated scales. It is also important to note that Guterman et al’s study [26] was conducted in three American states, and it is plausible that differences in culture, healthcare systems, and support services might explain this difference in findings.

The results indicated that caregiver-related sociodemographic variables such as education, household composition and whether the caregiver lived with the PWD, were not significantly related with any of the health-care utilization variables within the present study. There has been limited research regarding household composition and healthcare utilization. Although there has been some evidence that amongst larger households, care for the PWD may be distributed among a greater number of caregivers [46], it has also been suggested that most of the hands-on day-to-day care is often still left to a single individual [47]. These mixed findings may suggest that household composition can influence caregiver strain, but it appears not to have a direct association with healthcare utilization within the present sample. Similarly, although it has been posited that caregivers with higher education experienced increased burden [9, 48], it would appear that this association may not directly influence the healthcare utilization of PWD.

### Limitations and directions for future studies

The present study does contain some limitations that might limit the validity of its findings. Firstly, the healthcare utilization variables in the present study were based on self-report questionnaires by both PWD and caregivers and may thus be subject to recall bias. Moreover, the current study did not collect administrative data that could be used to cross-check this information or presented as an objective variable of interest. Secondly, the study did not include data on frailty and functional status of the PWD. Amongst older adult and PWD, frailty and unmet needs for help with basic activities of daily living have been shown to be strongly associated with increased healthcare utilization [49–51]. It would be pertinent for future studies adjust for these PWD-related factors. Third, Beeber et al. [52] found that the extent of the caregiver’s physical limitations due to comorbidities were associated with utilization of home-based support services (e.g. home care, adult day care, and respite care). Therefore, it would be prudent for future studies to examine how the caregiver’s physical health would also affect healthcare utilization of the PWD. Another variable of note that can also be examined is caregiver burnout, since previous studies have shown that it affects healthcare utilization of the PWD [45]. The findings of the present study might also be influenced by cultural factors. Given that Singapore is a multi-ethnic Asian society, it might be possible that cultural values such as filial piety of the caregivers might influence the reporting and experience of burden, distress, anxiety, and depression arising from the caregiving process. If these were underreported, it might have weakened the associations with healthcare utilization in the present paper. The data of the present paper was collected from 2012 to 2013 and thus it may not reflect the present situation regarding PWD and their caregivers. Nevertheless, the findings do point out vulnerabilities related to caregivers that need to be addressed. Lastly, the present study is of a cross-sectional nature, and therefore is limited in the ability to identify casual relationships between caregiver correlates and healthcare utilization.

## Conclusion

In conclusion, the study provided evidence that caregivers’ feelings of burden and distress related to responsive behaviours were associated with the healthcare utilization of PWD. The study therefore identifies vulnerabilities within caregivers of PWD and possible opportunities for intervention. For example, a recent publication suggested a staging strategy based upon symptoms of the PWD provided by the caregiver [53]. Using this information, subsequent interventions can be tailored to the different challenges faced by caregivers in different stages of dementia, thereby improving their ability to cope with the situation and reduce burden. Home-based interventions such as problem-solving therapy provided to caregivers are also effective in improving caregiver competence, burden, and perceived stress, which may then reduce healthcare utilization [54]. Healthcare providers and policy makers may choose to implement such interventions to alleviate the strain suffered by caregivers which will ameliorate the quality of life of both caregivers and PWD.

## Supplementary Information

**Additional file 1:.** Appendix

## Data Availability

The datasets used and/or analysed during the current study are available from the corresponding author on reasonable request.

## Abbreviations

BPSD: Behavioural and psychological symptoms
IRR: Incidence Rate Ratio
NPI-Q: Neuropsychiatric Inventory Questionnaire
OR: Odds Ratio
PWD: Persons with Dementia
SRQ-20: Self-Reporting Questionnaire
WiSE: Well-being of Singapore Elderly Study
ZBI: Zarit Burden Interview
