# Supplementary material for: The association between caregiver burden, distress, psychiatric morbidity and healthcare utilization among persons with dementia in Singapore
Source: BMC Geriatr. 2021 Jan 19;21:67. doi: 10.1186/s12877-021-02014-2 (PMC7816438; doi:10.1186/s12877-021-02014-2)
Supplement: Supplementary file 1 — Additional file 1:. Appendix [file 12877_2021_2014_MOESM1_ESM.docx]

**Appendix A**

| Table 1 | | | | | | | | | | | | | | |
| --- | --- | --- | --- | --- | --- | --- | --- | --- | --- | --- | --- | --- | --- | --- |
| Univariate analyses of variables with emergency room use | | | | | | | | | | | | | | |
|  | | | Emergency Room use | | | | | | | | | |  | |
|  | | | Yes  (n=356, 89.2%) | | | | | No  (n=43, 10.8%) | | | | |  | |
| Categorical variables | | | n | | % | | | n | | % | | | *p^a^* | |
| Age of Caregivers | | |  | |  | | |  | |  | | |  | |
| 21-49 | | | 98 | | 27.6% | | | 16 | | 37.2% | | | 0.41 | |
| 50-64 | | | 186 | | 52.4% | | | 20 | | 46.5% | | |  |  |
| 65 and above | | | 71 | | 20.0% | | | 7 | | 16.3% | | |  |  |
| Missing | | | 1 | | 0.3% | | | 0 | | 0 | | |  | |
| Gender | | |  | |  | | |  | |  | | |  | |
| Female | | | 236 | | 66.3% | | | 34 | | 79.1% | | | 0.10 | |
| Male | | | 119 | | 33.4% | | | 9 | | 20.9% | | |  |  |
| Missing | | | 1 | | 0.3% | | | 0 | | 0.0% | | |  | |
| Caregiver lives with participant | | |  | |  | | |  | |  | | |  | |
| No | | | 59 | | 16.6% | | | 7 | | 16.3% | | | 0.96 | |
| Yes | | | 296 | | 83.2% | | | 36 | | 83.7% | | |  |  |
| Missing | | | 1 | | 0.3% | | | 0 | | 0.0% | | |  | |
| Caregiver education | | |  | |  | | |  | |  | | |  | |
| None/Minimal | | | 37 | | 10.4% | | | 3 | | 7.0% | | | 0.50 | |
| Completed primary | | | 81 | | 22.8% | | | 7 | | 16.3% | | |  |  |
| Completed secondary | | | 144 | | 40.5% | | | 22 | | 51.2% | | |  |  |
| Completed tertiary | | | 93 | | 26.1% | | | 10 | | 23.3% | | |  |  |
| Missing | | | 1 | | 0.3% | | | 1 | | 2.3% | | |  | |
| Multimorbidity of PWD | | |  | |  | | |  | |  | | |  | |
| No Multimorbidity^┼^ | | | 147 | | 41.3% | | | 6 | | 14.0% | | | **< 0.001** | |
| Has Multimorbidity | | | 209 | | 58.7% | | | 37 | | 86.1% | | |  |  |
| Clinical dementia rating of PWD (CDR) | | |  | |  | | |  | |  | | |  | |
| No/Questionable | | | 103 | | 28.9% | | | 6 | | 14.0% | | | 0.11 | |
| Mild | | | 163 | | 45.8% | | | 25 | | 58.1% | | |  |  |
| Moderate and Severe | | | 90 | | 25.3% | | | 12 | | 27.9% | | |  |  |
| Participant receives income/ benefits/ pension/allowance | | |  | |  | | |  | |  | | |  | |
| No | | | 152 | | 42.7% | | | 19 | | 44.2% | | | 0.67 | |
| Yes | | | 203 | | 57.0% | | | 22 | | 51.2% | | |  |  |
| Missing | | | 1 | | 0.3% | | | 2 | | 4.7% | | |  | |
| Household composition | | |  | |  | | |  | |  | | |  | |
| Lives alone/With spouse only | | | 28 | | 7.9% | | | 3 | | 7.0% | | | 0.68 | |
| With spouse and others | | | 110 | | 30.9% | | | 16 | | 37.2% | | |  |  |
| With others | | | 212 | | 59.6% | | | 23 | | 53.5% | | |  |  |
| Missing | | | 6 | | 1.7% | | | 1 | | 2.3% | | |  | |
|  | | |  | |  | | |  | |  | | |  | |
| Continuous variables | | | | | | | | | | | | | | |
|  | Emergency room use | n | | Mean (S.D.) | | P25 | Median | | P75 | | Min | Max | | *p*^b^ |
| Caregiver distress from responsive behaviours (NPI-Q) | No | 343 | | 2.71 (5.3) | | 0 | 0 | | 3 | | 0 | 48 | | **0.001** |
|  | Yes | 43 | | 7.07(7.1) | | 0 | 3 | | 12 | | 0 | 31 | |  |
|  | Missing | 13 | | - | | - | - | | - | | - | - | |  |
| Psychiatric morbidity (SRQ-20) | No | 347 | | 2.3 (3.5) | | 0 | 1 | | 3 | | 0 | 20 | | **0.005** |
|  | Yes | 41 | | 3.7 (3.9) | | 0 | 2 | | 5 | | 0 | 16 | |  |
|  | Missing | 11 | | - | | - | - | | - | | - | - | |  |
| Zarit Burden Interview (ZBI) | No | 291 | | 16.4 (13.6) | | 6 | 13 | | 25 | | 0 | 88 | | 0.08 |
|  | Yes | 37 | | 21.7 (16.8) | | 7 | 18 | | 33 | | 0 | 64 | |  |
|  | Missing | 71 | | - | | - | - | | - | | - | - | |  |
| ^a^based on univariate chi-square analysis  ^b^based on univariate Mann-Whitney-U analysis  ^┼^The “No chronic conditions” and “At least one chronic condition” was subsumed into the “No Multimorbidity” group as no respondents in the “none” group endorsed having emergency room use | | | | | | | | | | | | | | |

**Appendix B**

| Table 2 | | | | | | | | | | | | | | |
| --- | --- | --- | --- | --- | --- | --- | --- | --- | --- | --- | --- | --- | --- | --- |
| Univariate analyses of variables with hospital admissions | | | | | | | | | | | | | | |
|  | | | Hospital Admissions | | | | | | | | | |  | |
|  | | | Yes  (n=347, 87.0%) | | | | | No  (n=52, 13.0%) | | | | |  | |
| Categorical variables | | | n | | % | | | n | | % | | | *p*^a^ | |
| Age of Caregiver | | |  | |  | | |  | |  | | |  | |
| 21-49 | | | 99 | | 28.5% | | | 15 | | 28.9% | | | 0.95 | |
| 50-64 | | | 180 | | 51.9% | | | 26 | | 50.0% | | |  |  |
| 65 and above | | | 67 | | 19.3% | | | 11 | | 21.2% | | |  |  |
| Missing | | | 1 | | 0.3% | | | 0 | | 0.0% | | |  | |
| Gender | | |  | |  | | |  | |  | | |  | |
| Female | | | 232 | | 66.9% | | | 38 | | 73.1% | | | 0.39 | |
| Male | | | 114 | | 32.9% | | | 14 | | 26.9% | | |  |  |
| Missing | | | 1 | | 0.3% | | | 0 | | 0 | | |  | |
| Caregiver lives with participant | | |  | |  | | |  | |  | | |  | |
| No | | | 59 | | 17.0% | | | 7 | | 13.5% | | | 0.52 | |
| Yes | | | 287 | | 82.7% | | | 45 | | 86.5% | | |  |  |
| Missing | | | 1 | | 0.3% | | | 0 | | 0.0% | | |  | |
| Caregiver education | | |  | |  | | |  | |  | | |  | |
| None/Minimal | | | 35 | | 10.1% | | | 5 | | 9.6% | | | 0.33 | |
| Completed primary | | | 78 | | 22.5% | | | 10 | | 19.2% | | |  |  |
| Completed secondary | | | 139 | | 40.1% | | | 27 | | 51.9% | | |  |  |
| Completed tertiary | | | 94 | | 27.1% | | | 9 | | 17.3% | | |  |  |
| Missing | | | 1 | | 0.3% | | | 1 | | 1.9% | | |  | |
| No Multimorbidity of PWD | | |  | |  | | |  | |  | | |  | |
| No Multimorbidity^┼^ | | | 145 | | 41.8% | | | 8 | | 15.4% | | | **< 0.001** | |
| Has Multimorbidity | | | 202 | | 58.2% | | | 44 | | 84.6% | | |  |  |
| Clinical dementia rating of PWD (CDR) | | |  | |  | | |  | |  | | |  | |
| No/Questionable | | | 102 | | 29.4% | | | 7 | | 13.5% | | | 0.053 | |
| Mild | | | 158 | | 45.5% | | | 30 | | 57.7% | | |  |  |
| Moderate and Severe | | | 87 | | 25.1% | | | 15 | | 28.9% | | |  |  |
| Participant receives income/ benefits/ pension/allowance | | |  | |  | | |  | |  | | |  | |
| No | | | 145 | | 41.8% | | | 26 | | 50.0% | | | 0.18 | |
| Yes | | | 201 | | 57.9% | | | 24 | | 46.2% | | |  |  |
| Missing | | | 1 | | 0.3% | | | 2 | | 3.9% | | |  | |
| Household composition | | |  | |  | | |  | |  | | |  | |
| Lives alone/With spouse only | | | 28 | | 8.1% | | | 3 | | 5.8% | | | 0.48 | |
| With spouse and others | | | 106 | | 30.6% | | | 20 | | 38.5% | | |  |  |
| With others | | | 207 | | 59.7% | | | 28 | | 53.9% | | |  |  |
| Missing | | | 6 | | 1.7% | | | 1 | | 1.9% | | |  | |
|  | | |  | |  | | |  | |  | | |  | |
| Continuous variables | | | | | | | | | | | | | | |
|  | Emergency room use | n | | Mean (S.D.) | | P25 | Median | | P75 | | Min | Max | | *p*^b^ |
| Caregiver distress from responsive behaviours (NPI-Q) | No | 335 | | 2.7 (5.3) | | 0 | 0 | | 3 | | 0 | 48 | | **0.003** |
|  | Yes | 51 | | 6.6 (9.2) | | 0 | 2 | | 11 | | 0 | 31 | |  |
|  | Missing | 13 | | - | | - | - | | - | | - | - | |  |
| Psychiatric morbidity (SRQ-20) | No | 338 | | 2.3 (3.4) | | 0 | 1 | | 3 | | 0 | 20 | | **0.01** |
|  | Yes | 50 | | 3.7 (4.2) | | 0 | 2 | | 6 | | 0 | 16 | |  |
|  | Missing | 11 | | - | | - | - | | - | | - | - | |  |
| Zarit Burden Interview (ZBI) | No | 283 | | 15.9 (13.3) | | 6 | 12 | | 24 | | 0 | 88 | | **0.004** |
|  | Yes | 45 | | 23.6 (17.2) | | 10 | 21 | | 35 | | 0 | 69 | |  |
|  | Missing | 71 | | - | | - | - | | - | | - | - | |  |
| ^a^based on univariate chi-square analysis  ^b^based on univariate Mann-Whitney-U analysis  ^┼^The “No chronic conditions” and “At least one chronic condition” was subsumed into the “No Multimorbidity” group as no respondents in the “none” group endorsed having hospital admission | | | | | | | | | | | | | | |

**Appendix C**

| Table 3 | | | | | | | | |
| --- | --- | --- | --- | --- | --- | --- | --- | --- |
| Univariate analyses with length of stay in hospital | | | | | | | | |
|  | Number of days spent in hospital (n=51) | | | | | | |  |
| Categorical variables | n | Mean (S.D.) | P25 | Median | P75 | Min | Max | *p*^a^ |
| Age group of caregivers |  |  |  |  |  |  |  |  |
| 21-49 | 15 | 7.1 (4.9) | 3 | 6 | 10 | 2 | 18 | 0.08 |
| 50-64 | 25 | 6.2 (19.9) | 3 | 5 | 9 | 1 | 17 |  |
| 65 and above | 11 | 27.8 (30.6) | 4 | 14 | 45 | 2 | 90 |  |
| Gender |  |  |  |  |  |  |  |  |
| Female | 37 | 9.4 (13.5) | 3 | 6 | 10 | 1 | 75 | 0.35 |
| Male | 14 | 15.8 (23.3) | 4 | 6.5 | 18 | 2 | 90 |  |
| Caregiver living with participant |  |  |  |  |  |  |  |  |
| No | 6 | 5.3 (2.73) | 3 | 4.5 | 7 | 3 | 10 | 0.49 |
| Yes | 45 | 11.9 (17.7) | 3 | 6 | 14 | 1 | 90 |  |
| Caregiver Education |  |  |  |  |  |  |  |  |
| None/Minimal | 5 | 11.4 (18.8) | 3 | 3 | 4 | 2 | 45 | 0.57 |
| Completed primary | 10 | 13.9 (21.9) | 5 | 6.5 | 9 | 2 | 75 |  |
| Completed secondary | 26 | 11.9 (17.7) | 4 | 6.5 | 14 | 1 | 90 |  |
| Completed tertiary | 9 | 6.4 (5.0) | 3 | 5 | 7 | 1 | 15 |  |
| Missing | 1 | - | - | - | - | - | - |  |
| Multimorbidity of PWD |  |  |  |  |  |  |  |  |
| No Multimorbidity^┼^ | 8 | 8.1 (9.5) | 2 | 4.5 | 10 | 2 | 30 | 0.48 |
| Has Multimorbidity | 43 | 11.7 (17.8) | 4 | 6 | 14 | 1 | 90 |  |
| Clinical dementia rating of PWD (CDR) |  |  |  |  |  |  |  |  |
| No/Questionable | 7 | 4.1 (3.1) | 2 | 4 | 6 | 1 | 10 | 0.11 |
| Mild | 29 | 12.69 (20.5) | 3 | 5 | 14 | 1 | 90 |  |
| Moderate and Severe | 15 | 11.33 (11.6) | 5 | 7 | 14 | 2 | 45 |  |
| Participant receives income/ benefits/ pension/allowance |  |  |  |  |  |  |  |  |
| No | 25 | 11.2 (15.4) | 4 | 6 | 14 | 2 | 75 | 0.78 |
| Yes | 24 | 11.1 (18.9) | 3 | 6 | 10 | 1 | 90 |  |
| Missing | 2 | - | - | - | - | - | - |  |
| Household composition |  |  |  |  |  |  |  |  |
| Lives alone/With spouse only | 3 | 12.3 (15.3) | 3 | 4 | 30 | 3 | 90 | 0.23 |
| With spouse and others | 19 | 17.4 (25.0) | 5 | 7 | 14 | 2 | 90 |  |
| With others | 28 | 7.0 (6.3) | 2 | 5 | 9.5 | 2 | 90 |  |
| Missing | 1 | - | - | - | - | - | - |  |
|  |  |  |  |  |  |  |  |  |
| Continuous variables | IRR | 95% CI | *p*^b^ |  |  |  |  |  |
| Caregiver distress from responsive behaviours (NPI-Q) | 0.99 | 0.97 – 1.03 | 0.92 |  |  |  |  |  |
| Psychiatric morbidity (SRQ-20) | **1.10** | 1.03 – 1.17 | **0.003** |  |  |  |  |  |
| Zarit Burden Interview (ZBI) | 1.02 | 1.01 – 1.04 | **0.005** |  |  |  |  |  |
| ^a^based on kruskal-wallis analyses  ^b^based on negative binomial analyses | | | | | | | | |

**Appendix D**

| Table 3 | | | | | | | | |
| --- | --- | --- | --- | --- | --- | --- | --- | --- |
| Univariate analyses of variables associated with number of outpatient visits | | | | | | | | |
|  | Number of outpatient visits (n=283) | | | | | | |  |
| Categorical variables | n | Mean (S.D.) | P25 | Median | P75 | Min | Max | *p*^a^ |
| Age group of caregivers |  |  |  |  |  |  |  |  |
| 21-49 | 81 | 2.5 (2.7) | 1 | 1 | 3 | 1 | 19 | 0.27 |
| 50-64 | 148 | 2.1 (2.7) | 1 | 1 | 2 | 1 | 29 |  |
| 65 and above | 53 | 1.7 (1.1) | 1 | 1 | 2 | 1 | 5 |  |
| Missing | 1 | - | - | - | - | - | - |  |
| Gender |  |  |  |  |  |  |  |  |
| Female | 190 | 2.2 (2.9) | 1 | 1 | 1 | 1 | 29 | 0.88 |
| Male | 92 | 1.9 (1.4) | 1 | 1 | 2 | 1 | 8 |  |
| Missing | 1 | - | - | - | - | - | - |  |
| Caregiver living with participant |  |  |  |  |  |  |  |  |
| No | 45 | 2.2 (1.6) | 1 | 1 | 3 | 1 | 8 | 0.42 |
| Yes | 237 | 2.1 (2.6) | 1 | 1 | 2 | 1 | 29 |  |
| Missing | 1 | - | - | - | - | - | - |  |
| Caregiver Education |  |  |  |  |  |  |  |  |
| None/Minimal | 25 | 1.8 (1.4) | 1 | 1 | 2 | 1 | 8 | 0.65 |
| Completed primary | 55 | 1.7 (1.1) | 1 | 1 | 2 | 1 | 6 |  |
| Completed secondary | 122 | 2.1 (2.3) | 1 | 1 | 3 | 1 | 19 |  |
| Completed tertiary | 79 | 2.5 (3.4) | 1 | 1 | 3 | 1 | 29 |  |
| Missing | 2 | - | - | - | - | - | - |  |
| Multimorbidity of PWD |  |  |  |  |  |  |  |  |
| No Multimorbidity^┼^ | 94 | 1.5 (0.9) | 1 | 1 | 2 | 1 | 5 | **< 0.001** |
| Has Multimorbidity | 189 | 2.4 (2.9) | 1 | 2 | 3 | 1 | 29 |  |
| Clinical dementia rating of PWD (CDR) |  |  |  |  |  |  |  |  |
| No/Questionable | 84 | 1.9 (1.5) | 1 | 1 | 2 | 1 | 8 | 0.17 |
| Mild | 137 | 2.3 (2.3) | 1 | 1 | 3 | 1 | 19 |  |
| Moderate and Severe | 62 | 2.1 (3.7) | 1 | 1 | 2 | 1 | 29 |  |
| Participant receives income/ benefits/ pension/allowance |  |  |  |  |  |  |  |  |
| No | 120 | 2.1 (2.8) | 1 | 1 | 2 | 1 | 29 | 0.52 |
| Yes | 160 | 2.2 (2.2) | 1 | 1 | 3 | 1 | 19 |  |
| Missing | 3 | - | - | - | - | - | - | - |
| Household composition |  |  |  |  |  |  |  |  |
| Lives alone/With spouse only | 23 | 1.6 (1.0) | 1 | 1 | 2 | 1 | 5 | 0.11 |
| With spouse and others | 89 | 2.7 (3.8) | 1 | 2 | 3 | 1 | 29 |  |
| With others | 166 | 1.9 (1.5) | 1 | 1 | 2 | 1 | 10 |  |
| Missing | 5 | - | - | - | - | - | - | - |
|  |  |  |  |  |  |  |  |  |
| Continuous variables | IRR | 95% CI | *p*^b^ |  |  |  |  |  |
| Caregiver distress from responsive behaviours (NPI-Q) | 1.01 | 0.99 – 1.02 | 0.30 |  |  |  |  |  |
| Psychiatric morbidity (SRQ-20) | 0.99 | 0.97 – 1.02 | 0.65 |  |  |  |  |  |
| Zarit Burden Interview (ZBI) | **1.01** | 1.002 – 1.02 | **0.01** |  |  |  |  |  |
| ^a^based on kruskal-wallis analyses  ^b^based on negative binomial analyses | | | | | | | | |
